# The TNF‐α‐induced expression of miR‐130b protects cervical cancer cells from the cytotoxicity of TNF‐α

**DOI:** 10.1002/2211-5463.12395

**Published:** 2018-02-16

**Authors:** Lei Yang, Yanli Wang, Shuainan Shi, Lili Xie, Tao Liu, Yuliang Wang, Hong Mu

**Affiliations:** ^1^ Department of Clinical Laboratory Tianjin First Center Hospital China; ^2^ Department of Clinical Laboratory Tianjin Hospital of ITCWM Nankai Hospital China; ^3^ First Center Clinical College Tianjin Medical University China; ^4^ Key Laboratory for Critical Care Medicine of the Ministry of Health Tianjin China

**Keywords:** cervical cancer cell, miR‐130b, PTEN, TNF‐α resistance

## Abstract

Tumour necrosis factor alpha (TNF‐α) is a multifunctional cytokine and has the capacity both to promote cell growth and to kill tumour cells by inducing cell apoptosis. However, many tumour cells develop resistance to the toxic effects of TNF‐α. Thus, understanding the mechanism underlying the resistance of tumours to TNF‐α toxicity and finding ways to overcome this resistance are urgently needed. In this study, we discovered that two cervical cancer cell lines, Hela and Siha, showed null responses to TNF‐α cytotoxicity. However, in these cell lines, TNF‐α stimulation promoted the expression of miR‐130b and downregulated the expression of *PTEN* gene, which encodes a dual‐specificity phosphatase that acts as a tumour suppressor. Blockade of miR‐130b function or overexpression of *PTEN* gene sensitized cells to TNF‐α cytotoxicity. Regression analyses revealed that there were reverse relationships between the cellular levels of miR‐130b and *PTEN* mRNA in cervical cancer cells. Gain‐ and loss‐of‐function assays demonstrated that there were causal relationships between the increase in miR‐130b levels and the reduction in *PTEN* mRNA or PTEN protein levels. *In silico* analysis revealed that there were two miR‐130b target sites within the 3′UTR of *PTEN* mRNA and experimental evidences demonstrated that miR‐130b repressed the expression of *PTEN* gene by binding directly to the 3′UTR of *PTEN* mRNA. These data suggest miR‐130b expression as a target to be inhibited to make tumour cells more sensitive to the toxic impact of TNF‐α.

**Abbreviations**
2′ OMe: 2′‐O‐methylation
cDNA: complementary DNA
DMSO: dimethyl sulfoxide
FITC: fluorescein isothiocyanate
GAPDH: glyceraldehyde‐3‐phosphate dehydrogenase
GFP: green fluorescent protein
miRNA: microRNA
NF‐κB: nuclear factor kappa‐light‐chain‐enhancer of activated B cells
NS: nonsignificant
PI: propidium iodide
PTEN: phosphatase and tensin homolog deleted on chromosome ten
SE: standard error
TBST: Tris‐buffered saline with Tween‐20
TNF‐α: tumour necrosis factor alpha
UTR: untranslated region

Tumour necrosis factor alpha (TNF‐α) is a 26‐kilodalton (kDa) type II transmembrane protein, which is arranged in a stable homotrimers. Its soluble homotrimeric cytokine is derived from the transmembrane form through proteolytic cleavage by the metalloprotease TNF‐α converting enzyme 1. In the case of cancer development, TNF‐α is a double‐edged sword. On the one hand, it could act as an endogenous tumour promoter based on its abilities to stimulate the growth, proliferation, invasion and metastasis of cells 2, 3, 4. On the other hand, it performs as a cancer killer relying on its capacity of inducing cell apoptosis 5. The property of TNF‐α in promoting the death of cancer cell suggests TNF‐α to be a potential cancer therapeutic drug. Unfortunately, the cytotoxic effect of TNF‐α is cell line specific and only a proportion of tumour cell lines are sensitive to TNF‐α cytotoxicity 6. Therefore, understanding the mechanism underlying the resistance of tumour cells to TNF‐α killing and finding available approaches to overcome the drug resistance are urgently required.

MicroRNA (miRNA) refer to a class of small noncoding RNA of approximately 20 nucleotides in length. They play important roles in gene regulations in animals’ cells by base pairing with the UTR of their target genes 7, 8. It was previously recorded that the upregulated expression of miR‐19a in colorectal cancer cell responded to TNF‐α stimulation‐mediated TNF‐α‐induced epithelial‐to‐mesenchymal transition of the tumour cell 9. The expression of miR‐146a induced by TNF‐α‐inhibited TNF‐α‐induced adipogenesis via targeting insulin receptor 10. Wei R and his coworkers found that the downregulated expression of miR‐23a caused by TNF‐α stimulation led to an increase in endothelial cell apoptosis induced by TNF‐α 11. Suarez Y and his fellows discovered that TNF‐α enhanced the expression of miR‐31 and miR‐17‐3p but the upregulated miRNA could antagonized the increased expression of E‐selectin and intercellular adhesion molecules‐1 (ICAM‐1) triggered by TNF‐α 12. These literatures illustrated that TNF‐α‐induced miRNA could provide positive or negative feedback controls of the impacts of TNF‐α on cell phenotypes by modulating the expression of their specific targeted genes.

The biological functions of miR‐130b in the development of tumours were inconsistent in different cell lines. On the one hand, miR‐130b made a negative effect on the development of some tumours. It inhibited cell proliferation and invasion in pancreatic cancer cell through downregulating STAT3 expression and suppressed the migration and invasion of colorectal cancer cell by decreasing the expression of integrin β1 13, 14. On the other hand, the miRNA affected the tumour progression in a positive manner in other specific cells. In oesophageal squamous cell carcinoma cell, it worked as an oncogenic role by inhibiting the expression of *PTEN* gene 15. The upregulation of miR‐130b expression contributed to the development of thyroid adenomas by targeting CCDC6 gene 16. MiR‐130b could promote cell migration and invasion by decreasing the *PTEN* gene expression through FAK and Akt phosphorylation in bladder cancer 17. By inhibiting the expression of peroxisome proliferator‐activated receptor‐γ, miR‐130b could promote the proliferation and invasion of human glioma cell 18. As far as we know, there is lack of the research on the role of miR‐130b in the cervical cancer cell line. In this study, we found that the expression of miR‐130b was promoted by TNF‐α treatment in cervical cancer cell but the increased expression of miR‐130b affected the TNF‐α cytotoxicity in a negative way. We tried to gain insight to the mechanism underlying this phenomenon through our study and demonstrated that the inhibition of miR‐130b function enhanced the TNF‐α‐induced cell death of cervical cancer cell.

## Materials and methods

### Cell culture and TNF‐α treatment

Hela and Siha cells were planted in RPMI1640 medium supplemented with 10% (vol/vol) fetal bovine serum and 1% penicillin–streptomycin (vol/vol) and cultured in a humidified atmosphere containing 5% CO_2_ at 37 °C. The cultured cell was treated with either vehicle control formed by phosphate‐buffered saline with 1 mg·mL^−1^ bovine serum albumins (Sigma, St. Louis, MO, USA) or TNF‐α solution of wanted concentration which was prepared using commercial stock solution of TNF‐α (Sigma) for the desired incubation time period.

### RNA extraction

RNA was extracted from the cultured cell using RNA Isolation kit (Ambion, Inc, Austin, TX, USA). The quality assessment of isolated RNA was achieved through the analysis of integrity using gel electrophoresis and that of purity by calculating the ratio between RNA absorbance at 260 nm and its absorbance at 280 nm.

### The construction of vectors

The complementary DNA (cDNA) derived from a part of *PTEN* mRNA was synthesized within a vial containing SpnRT as a specific primer and reverse transcriptase following the manufacturer's instruction (Takara, Minato‐ku, Tokyo, Japan). The DNA fragment encompassing the coding region of *PTEN* gene and Kozak sequence ahead of the coding region was obtained and amplified through PCR using one pair of primers, ptnF1 and ptnR1, in addition to the other pair of primers, ptnF2 and ptnR2. The amplification products were digested using restriction enzyme (Thermo Scientific, Waltham, MA, USA), BamHI and EcoRI, and linked into the corresponding cleavage sites within pcDNA3.1 vectors to form pcDNA3.1::*PTEN* vectors using T4 DNA ligases (Thermo Scientific). A part of *PTEN* mRNA bearing predicted sites targeted by miR‐130b was reverse transcribed using a specific primer, SputrRT. Then, the two DNA fragments which encoded the parts of UTR of *PTEN* mRNA containing predicted targeted sites were separately amplified using two pairs of primers, putrU1 and putrD1 or putrU2 and putrD2, and then cloned into pEGFP vectors downstream of GFP coding domains to construct fusion vectors, pEGFP::wt1‐UTR and pEGFP::wt2‐UTR. The sequence complementary to the seed region of miR‐130b either in position 2654–2661 or 4495–4502 of the *PTEN* mRNA, TTGCACT, was mutated to the sequence, TAGGAGT, using two additional pairs of primers, pnmutU1 and pnmutD1 or pnmutU2 and pnmutD2, based on the site‐directed mutagenesis, respectively. The constructed vector containing mutated points within position 2654–2661 of the *PTEN* mRNA was named as pGFP::mut1‐UTR and the vector containing points within position 4495–4502 as pGFP::mut2‐UTR. The DNA sequences of all the available primers were listed in Table 1.

**Table 1:** The primers used in the construction of vectors

Name	Sequence
SpnRT	5′‐CATTTTCAGTTTATTCAAGTT‐3′
ptnF1	5′‐CCGCCACGATGGCAGCCATCATC‐3′
ptnR1	5′‐TTCCGCGAGCTCTCAGACTTTTGTAATTTGTG‐3′
ptnF2	5′‐CGGAATTCGCCGCCACGATGGC‐3′
ptnR2	5′‐CCGCTCGAGTTCCGCGAGCTCTCAG‐3′
SputrRT	5′‐ATAATGCCATTTTTCCAT‐3′
putrU1	5′‐CCGGAATTCAGACAGACTGATGTGTATACGTAG‐3′
putrD1	5′‐CGGGATCCTCTGAGCATTCCCTCC‐3′
putrU2	5′‐CCGGAATTCTATGCCACCTTGTCTTTCAT‐3′
putrD2	5′‐CGGGATCCCCAATGACTACACCATAAAAT‐3′
pnmutU1	5′‐TCCTACCCCTTAGGAGTTGTGGCAACAG‐3′
pnmutD1	5′‐TGTTGCCACAACTCCTAAGGGGTAGGAT‐3′
pnmutU2	5′‐ATGGGCTTTAGGAGTGTTATTATTTTTCCTTTGG‐3′
pnmutD2	5′‐AAAGGAAAAATAATAACACTCCTAAAGCCCATTATAATG‐3′

The underlined words represented the sequences recognized by restriction enzymes.

### Semiquantitative real‐time PCR assays

The RNA reverse transcriptions were achieved using reverse transcriptase following the product's manual (Takara). Real‐time PCR assays were performed using cDNA generated in the reverse transcription assays containing 50 ng of total RNA templates to quantify the relative levels of the miR‐130b according to the method described in the previous literature 19 and in the assays containing 200 ng of total RNA templates to determine the relative contents of *PTEN* mRNA using the protocol from a qRT‐PCR mRNA detection kit (Roche, Indianapolis, IN, USA). Amplifications and measurements of specific products were performed on a Roche Lightcycler 480 Detection System. U6 small RNA were employed as internal controls for miRNA templates normalization and β‐actin mRNA for *PTEN* templates normalization. The relative expression levels of RNA molecules in either TNF‐α‐treated cells or vehicle‐treated cells were calculated using 2^−ΔΔCT^ method 20. The sequences of the primers utilized in the reverse transcription or real‐time PCR assays were listed in Table 2.

**Table 2:** The primers used in the semiquantitative real‐time PCR assays

Name	Sequence
SpnRT^a^	5′‐CATTTTCAGTTTATTCAAGTT‐3′
OligodT_18_ b	5′‐TTTTTTTTTTTTTTTTTT‐3′
miRT^c^	5′‐GTCGTATCCAGTGCAGGGTCCGAGGTATTCTGCACTGGATACGACATGCCC‐3′
U6spRT^d^	5′‐TCACGAATTTGCGTGT‐3′
pnRetF^e^	5′‐GCCACAGGCTCCCAGACAT‐3′
pnRetR^e^	5′‐GCAGGAAATCCCATAGCAATAAT‐3′
bacRetF^f^	5′‐AGTTGCGTTACACCCTTTCTTG‐3′
bacRetR^f^	5′‐TGTCACCTTCACCGTTCCAGT‐3′
miRU^g^	5′‐AGTGCAGGGTCCGAGGTAT‐3′
miRD^g^	5′‐TGCAATGATGAAAGGGCAT‐3′
U6F^h^	5′‐CGCTTCGGCAGCACAT‐3′
U6R^h^	5′‐ATTTGCGTGTCATCCTTGC‐3′

^a^The primer was used to synthesize *PTEN* cDNA in the reverse transcription assay. ^b^The primer was used to synthesize β‐actin cDNA in the reverse transcription assay. ^c^The primer was used to synthesize miR‐130b cDNA in the reverse transcription assay. ^d^The primer was used to synthesize the cDNA of U6 small RNA in the reverse transcription assay. ^e^The pair of primers was used to amplify *PTEN* cDNA in the real‐time PCR assay. ^f^The pair of primers was used to amplify β‐actin cDNA in the real‐time PCR assay. ^g^The pair of primers was used to amplify miR‐130b cDNA in the real‐time PCR assay. ^h^The pair of primers was used to amplify the cDNA of U6 small RNA in the real‐time PCR assay.

### Transfection of cervical cancer cells

The transfection of tumour cell with exogenous nucleotides was accomplished using Xfect transfection reagent according to the guideline provided by the manufacturer (Takara). Briefly, the oligonucleotides including the miR‐130b mimics, miR‐130b inhibitors and their respective scrambled control oligonucleotides were utilized at final concentrations of 50 pmol·mL^−1^ and the plasmids including pcDNA3.1, pEGFP, rTagRFP and their respective derived plasmids were used at final concentrations of 3 μg·mL^−1^ in the transfection assays unless otherwise noted. The miR‐130b mimics are short double‐stranded RNA. Each molecule is constituted by one guide strand whose sequence is identical to the sequence of mature miR‐130b and one passenger stand partially complementary to the mature miR‐130b sequence. The sequence of the guide strand is ‘CAGUGCAAUGAUGAAAGGGCAU’, and the sequence of the passenger strand is ‘GCCCUUUCAUCAUUGCACUGUU’. The miR‐130b inhibitors are single‐stranded RNA with 2′‐O‐Methyl (2′OMe) modifications. Their sequences are complementary to the mature miR‐130b sequences and presented as ‘AUGCCCUUUCAUCAUUGCACUG’.

### MTT reduction assays

Cancer cells were seeded on a 96‐well plate at a density of 7000 cells per well and incubated in the medium overnight. On the next day, the mediums were removed and replaced by the mediums containing TNF‐α at indicated concentrations. About 20 μL of MTT solution (5 mg·mL^−1^) was added to the medium at desired time point after the treatment of cell with TNF‐α, and then, cells were incubated at 37 °C for additional 4 h. Another 100 μL of DMSO was supplied to each well to dissolve the purple formazan crystals after the removal of MTT solution, and the plate was shaken at room temperature over a time course of 20 min. The optical density was detected at 570 nm using an EnSpire™ Multilabel Reader (PerkinElmer, Wallac Oy, Turku, Finland). The viabilities of cells cultured in the medium free of TNF‐α were used as controls and considered as 100% of viabilities. Each group was composed of three independent assays.

### The measurements of cell apoptosis

The cervical cancer cells were labelled using Annexin‐V apoptosis kit according to the manufacturer's instruction (BD BioSciences, San Jose, CA, USA). The percentages of the apoptotic cells were determined using BD FACSCalibur Flow Cytometry System (BD BioSciences).

### Western blot assays

The lysis of cervical cancer cell was achieved in RIPA buffer (Solarbio Science, Beijing, China) following the instruction provided by the manufacturer. The protein concentrations in the extracts were determined using BCA Protein Assay Kit (Takara). The extracts containing 30 μg of total proteins were mixed with commercial 5× protein loading buffer (Bosterbio, Pleasanton, CA, USA) and then subjected to 10% SDS/PAGE. After the proteins electroblotted to PVDF membrane (Bosterbio), the membrane was blocked with 5% nonfat milk in Tris‐buffered saline with Tween‐20 (TBST) buffer. The membrane was then incubated with mouse anti‐PTEN or anti‐GAPDH antibody (1 : 200 dilution; Bosterbio) at 4 °C overnight. The membrane which was bound by anti‐PTEN or anti‐GAPDH antibody was washed three times with TBST buffer and then incubated with horseradish peroxidase‐conjugated rabbit anti‐mouse IgM (1 : 2000 dilution; Bosterbio) for 2 h. After that, the membrane was washed three times with TBST buffer once again. The protein contents were shown using an enhanced chemiluminescence detection kit (Bosterbio) and analysed using alphaview sa software (ProteinSimple, Santa Clara, CA, USA). The band intensities of GADPH proteins were utilized as internal controls for the normalization of PTEN protein levels.

### Statistic analysis

All data were analysed using graphpad prism 5.0 software (GraphPad Software Inc, San Diego, CA, USA). The numerical data were calculated and described as means ± SEM. To determine the relative expression level of target gene, the mean value of the data obtained in control group was defined as 1 or 100%. Student's two‐tailed unpaired *t*‐tests were used for statistical evaluations of the data. The regression analysis was used to determine the relationship between two parameter sets. *P* values < 0.05 were considered to be significant.

## Results

### Cervical cancer cells developed resistance to cytotoxicity mediated by TNF‐α

**Figure 1:**
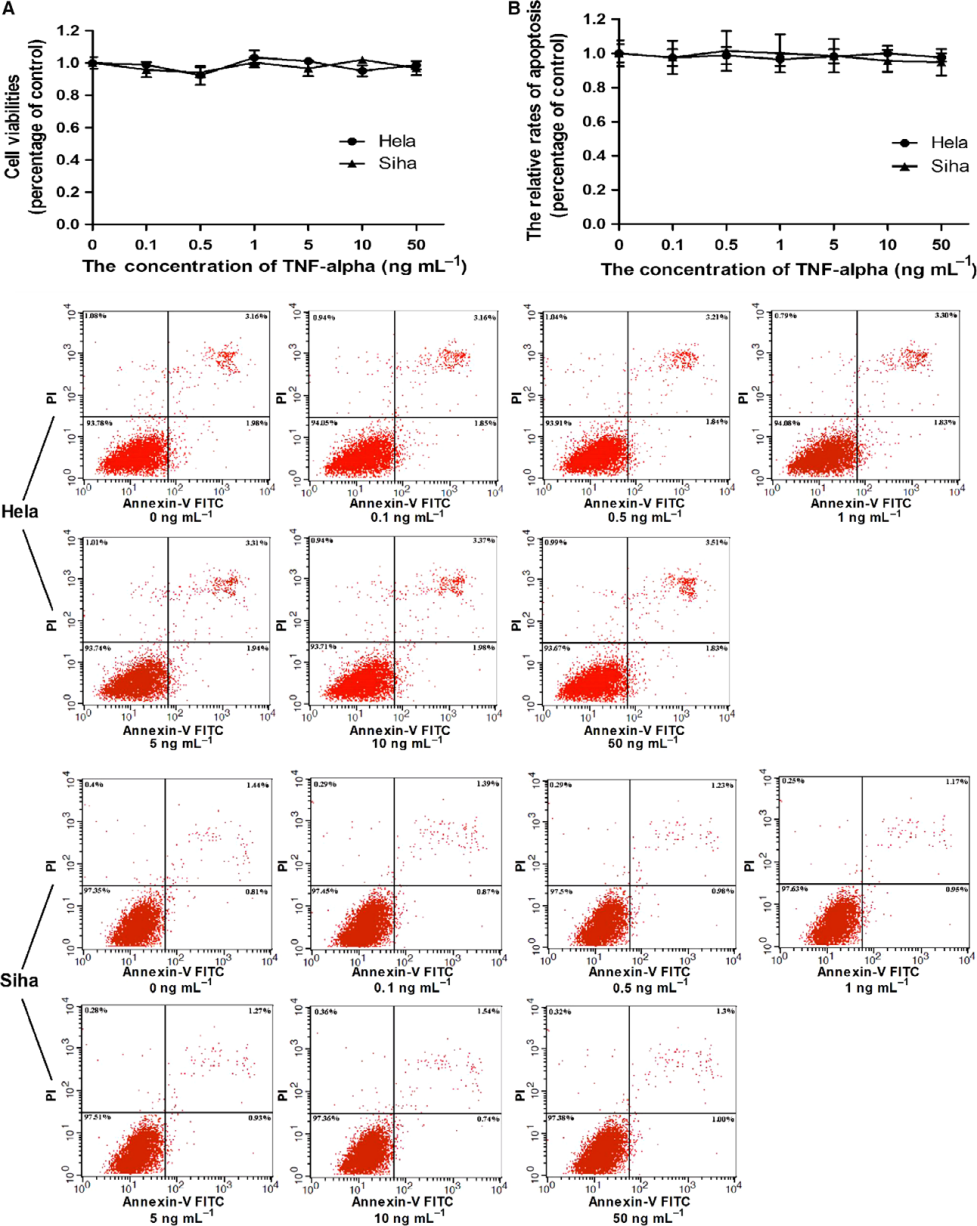
Cervical cancer cell developed resistance to TNF‐α toxic action. Cells were cultured in 96‐well plates with each well filled with the medium containing TNF‐α at various concentrations ranging from 0 to 50 ng·mL^−1^. The viabilities and apoptosis rates of target cells were determined by MTT assays and on the Flow Cytometry System, respectively. (A) There was no significant influence of TNF‐α stimulation on viabilities of Hela and Siha cells. (B) No obvious impact of TNF‐α treatment on apoptosis rate of either Hela or Siha cell was observed. Circles represented the relative levels of cell viabilities or apoptosis rates of TNF‐α‐treated Hela cells, and triangles represented the levels of treated Siha cells as compared with the corresponding levels of vehicle‐treated cells. Data were described as means ± SE (*n* = 3). The bars indicated the deviations from means. The graphic data describing the apoptosis rates of tumour cells located at the bottom of the statistical analyses of the graphic data and the concentrations of TNF‐α used in treating tumour cells were noted at the bottom of graphic data. The horizontal axis in graphic datum presented the fluorescence intensity of FITC which was conjugated with Annexin‐V and the vertical axis in datum indicated the intensity of propidium iodide (PI) which was used as a DNA stain.

To know the effects of TNF‐α on the viabilities of cervical cancer cells, Hela and Siha cells were incubated with TNF‐α at various concentrations and their viabilities were determined by MTT assays. As shown in Fig. 1A, there was no significant alteration among the viabilities of either Hela or Siha cells over 48‐h incubation time periods. Additionally, it is well known that TNF‐α shows its cytotoxicity by inducing apoptosis in a variety of cell types. To examine the impacts of TNF‐α on the apoptosis rates of Hela and Siha cells, the target cells were cultured in the medium containing TNF‐α at desired concentrations for 48‐h time periods. As expected, there was no obvious change among the apoptosis rates of target cells after exposure to TNF‐α (Fig. 1B).

### The TNF‐α‐induced expression of miR‐130b protected cervical cancer cell from toxic activity of TNF‐α

**Figure 2:**
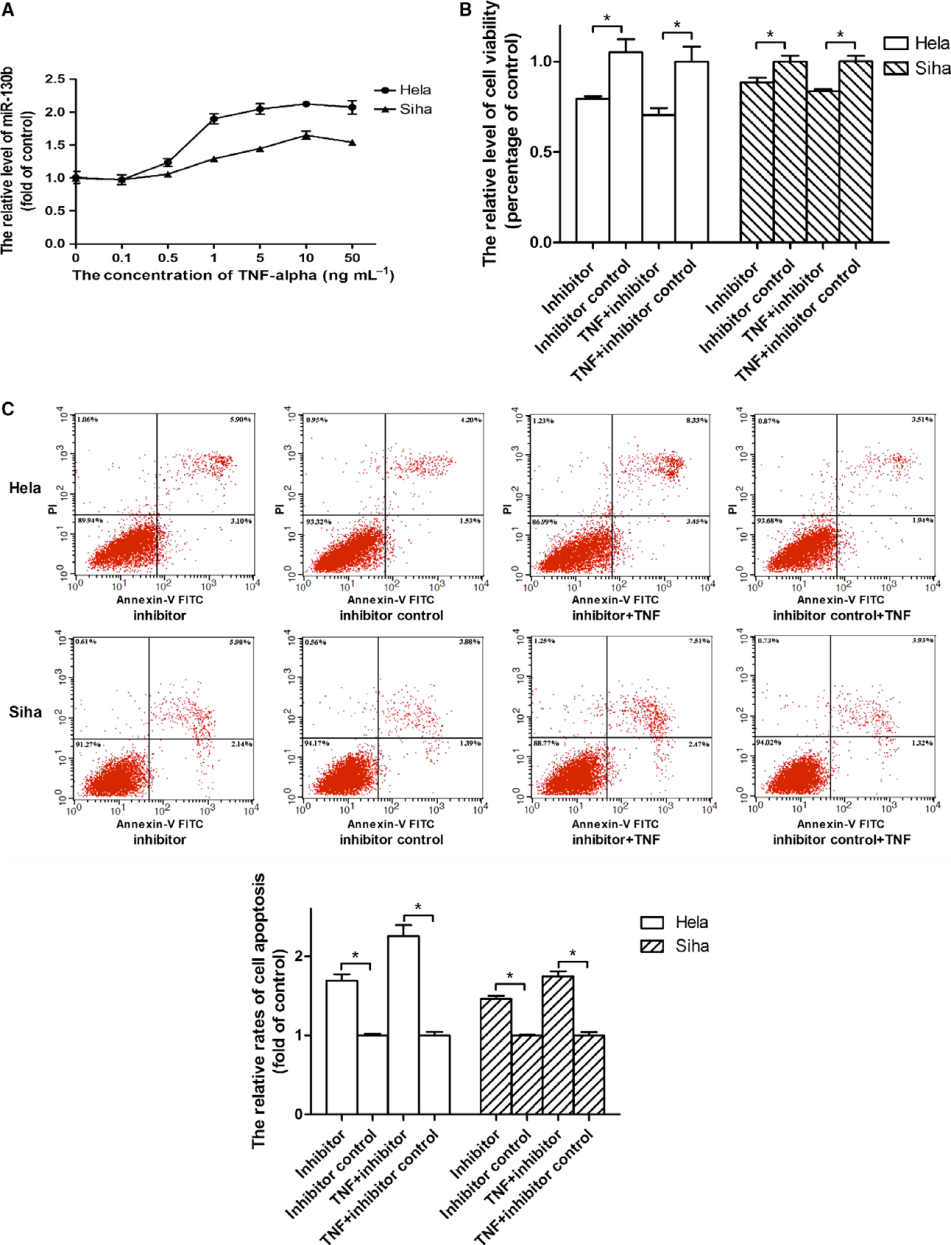
The TNF‐α‐induced expression of miR‐130b desensitized cervical cancer cell to toxic activity of TNF‐α. Cells were incubated with TNF‐α at indicated concentrations, and the relative expression levels of miR‐130b in target cells were measured in semiquantitative real‐time RT‐PCR assays at 48 h post‐treatments. (A) The incubation of Hela and Siha cells with TNF‐α elevated the expression levels of miR‐130b. Circles represented the relative levels of miR‐130b in TNF‐α‐treated Hela cells and triangles represented the levels in treated Siha cells as compared with their counterparts in vehicle‐treated cells. (B) The transfection of miR‐130b inhibitors decreased viabilities of TNF‐α‐treated Hela and Siha cells which were determined in MTT assays. (C) The transfection of miR‐130b inhibitors increased cell apoptosis rates of TNF‐α‐treated Hela and Siha cells. The note ‘inhibitor’ or ‘inhibitor control’ under the horizontal axis indicated the tumour cell transfected with miR‐130b inhibitor or its scrambled control oligonucleotide while ‘TNF‐α + inhibitor’ or ‘TNF‐α + inhibitor control’ indicated the cell with miR‐130b inhibitor or its scrambled control oligonucleotide prior to TNF‐α treatment. The graphic data describing the apoptosis rates of tumour cells located above the statistical analyses of the graphic data, and the types of oligonucleotides used in the transfection assays were described at the bottom of the graphic data. The blank columns represented the relative levels of cell viabilities or apoptosis rates of Hela cells, and the shadow ones represented the levels or rates of Siha cells. Data were described as means ± SE (*n* = 3). The bars indicated the deviations from means. The horizontal axis in graphic datum presented the fluorescence intensity of FITC which was conjugated with Annexin‐V, and the vertical axis in datum indicated the intensity of propidium iodide (PI) which was used as a DNA stain. *P < 0.05; NS, nonsignificant.

The TNF‐α‐induced expressions of miR‐130b have been described in both adipocyte and bladder carcinoma cell lines 21, 22. Through the analyses on the data from semiquantitative real‐time RT‐PCR assays, we found that the increment in the expression level of miR‐130b was in correlation with the elevation in the concentration of TNF‐α utilized to treat cell when the concentration of TNF‐α increased from 0 up to 10 ng·mL^−1^. The contents of miR‐130b increased by 1.09‐ and by 0.67‐fold in Hela and Siha cells after incubated with 10 ng·mL^−1^ TNF‐α over 48‐h time periods as compared with the corresponding contents in cells after incubated with vehicle (Fig. 2A). To investigate the impact of miR‐130b level on the cytotoxicity of TNF‐α to target cell, miR‐130b inhibitors were transfected into Hela or Siha cells to block the functions of endogenous miR‐130bs. After a 48‐h incubation time period, the transfection of miR‐130b inhibitors resulted in a 24.42% decrease in the viability of Hela and a 11.57% decrease in Siha cell cultured in the medium in the absence of TNF‐α but did led to a 29.57% reduction in the viability of Hela and a 16.33% reduction in Siha cell cultured in the medium containing 10 ng·mL^−1^ TNF‐α as compared with the transfection of the scrambled control oligonucleotides, respectively (Fig. 2B). To determine the impacts of inhibitors on the apoptosis rates of cervical cancer cells, miR‐130b inhibitors and their scrambled control oligonucleotides were employed at final concentrations of 100 pmol·mL^−1^ in the transfection assays. The transfection of inhibitors increased the apoptosis rate of Hela cell by 0.74‐fold and the rate of Siha cell by 0.46‐fold when the cell was cultured in the medium without TNF‐α but produced a 1.25‐fold increment in apoptosis rate of Hela and a 0.69 fold increment of Siha cell when the cell was cultured in the medium involving 50 ng·mL^−1^ TNF‐α as compared with the transfection of the scrambled control oligonucleotides, respectively (Fig. 2C).

### The suppressed expression of PTEN gene caused by TNF‐α stimulation rendered cervical cancer cells resistant to TNF‐α toxic actions

**Figure 3:**
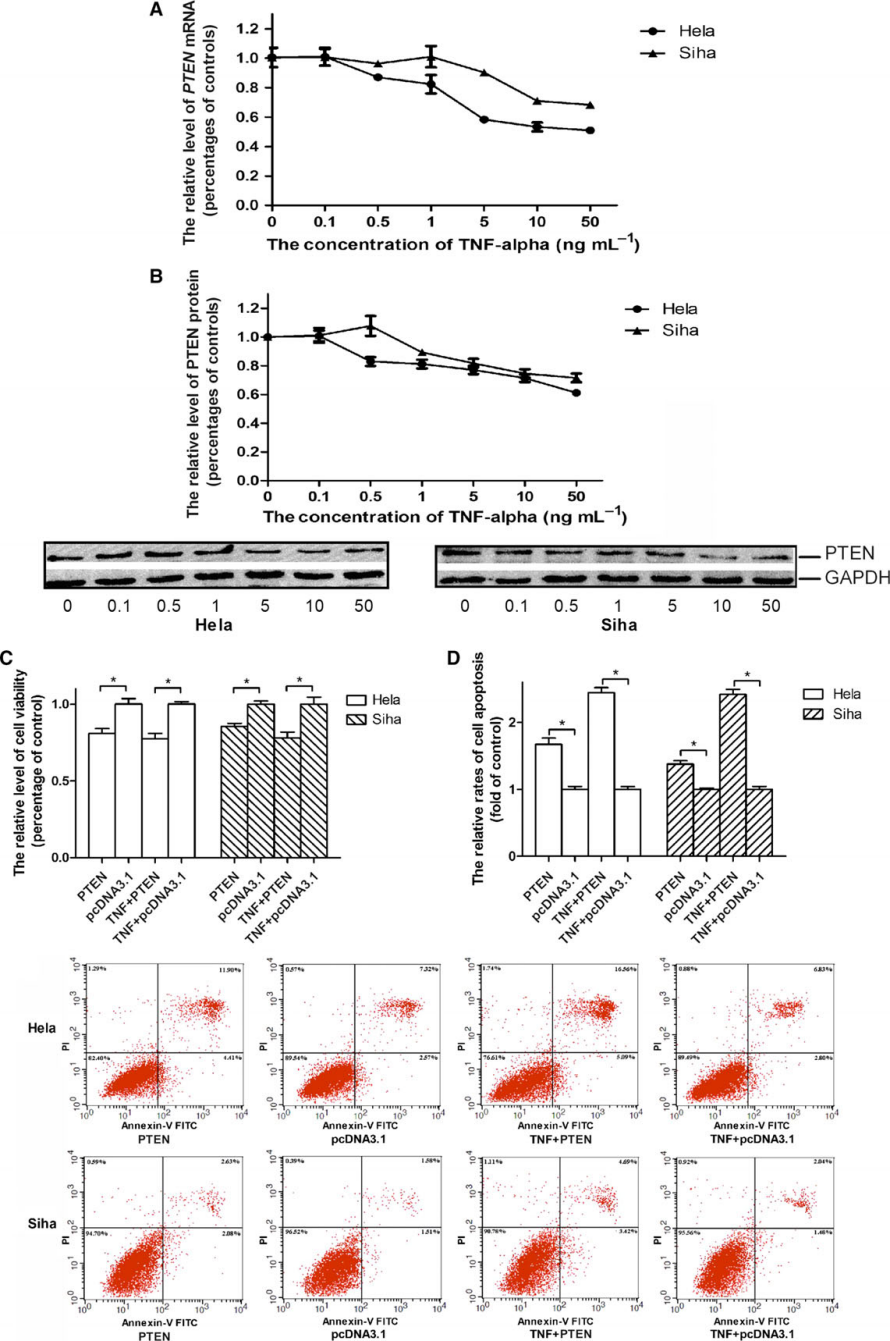
The suppression of *PTEN* gene caused by TNF‐α treatment protected cervical cancer cell to cytotoxicity of TNF‐α. Cells were incubated with TNF‐α at wanted concentrations over 48‐h time courses, and the relative expression levels of *PTEN* mRNA and proteins in target cells were determined in semiquantitative real‐time RT‐PCR and western blot assays. (A) The incubation of Hela and Siha cells with TNF‐α reduced the expression levels of *PTEN* mRNA. (B) The incubation of Hela and Siha cells with TNF‐α reduced the expression levels of PTEN proteins. The graphic data obtained in western blot assays located at the bottom of the statistical analyses of the graphic data. Circles represented the relative levels of *PTEN* mRNA or PTEN proteins in TNF‐α‐treated Hela cells, and triangles represented the levels in treated Siha cells as compared with the corresponding levels in vehicle‐treated cells. (C) The overexpression of *PTEN* gene in Hela or Siha cell decreased viability of the TNF‐α‐treated tumour cell. (D) The overexpression of *PTEN* gene in Hela or Siha cell promoted cell apoptosis of TNF‐α‐treated tumour cell. The note ‘PTEN’ or ‘pcDNA3.1’ under the horizontal axis indicated the tumour cell transfected with pcDNA3.1::*PTEN* or pcDNA3.1 while ‘TNF‐α + PTEN’ or ‘TNF‐α + pcDNA3.1’ indicated the cell with pcDNA3.1::*PTEN* or its control vector prior to TNF‐α treatment. The blank columns represented the relative levels of cell viabilities or apoptosis rates of Hela cells, and the shadow ones represented the levels or rates of Siha cells. Data were described as means±S. E. (*n* = 3). The bars indicated the deviations from means. The graphic data describing the apoptosis rates of tumour cells located at the bottom of the statistical analyses of the graphic data, and the types of the oligonucleotides used in the transfection assays were noted at the bottom of the graphic data. The horizontal axis in graphic datum presented the fluorescence intensity of FITC, which was conjugated with Annexin‐V, and the vertical axis in datum indicated the intensity of propidium iodide (PI), which was used as a DNA stain. *P < 0.05; NS, nonsignificant.

TNF‐α has been previously recorded to suppress the expression of *PTEN* gene in human colon cell lines 23. In our research, we got the consistent result in cervical cancer cell. The reductions in the cellular levels of *PTEN* mRNA as well as the levels of PTEN proteins in the Hela and Siha cells were accompanied with the increases in the concentrations from 0 to 50 ng·mL^−1^ of TNF‐α (Fig. 3A,B). To explore the impact of *PTEN* gene expression on the action of TNF‐α to the cervical cancer cell, pcDNA3.1::*PTEN* was transfected into Hela or Siha cell. When the post‐transfected cell was incubated in the medium lacking of TNF‐α over a 48‐h time period, pcDNA3.1::*PTE*N caused a 19.15% decrease in the viability of Hela cell and a 14.61% decrease in Siha cell, but when the target cell was incubated in the medium containing 10 ng·mL^−1^ TNF‐α for the same time period, pcDNA3.1::*PTEN* led to a 22.37% decrease in the viability of Hela cell and a 21.90% decrease in Siha cell when compared with its control vector (Fig. 3C). To explore the impacts of *PTEN* gene expressions on the apoptosis rates of cervical cancer cells, pcDNA3.1::*PTEN* vectors and their control vectors were used at final concentrations of 5 μg·mL^−1^ in the transfection assays. After the transfected target cells subjected to the same treatments as described above, the apoptosis rates of Hela and Siha cells transfected with pcDNA3.1::*PTEN* increased by 0.67‐ and 0.02‐fold in the absence of TNF‐α stimulation but elevated by 1.44‐ and 0.25‐fold in the presence of TNF‐α stimulation (Fig. 3D).

### MiR‐130b mediated the suppression of PTEN expression caused by TNF‐α treatment

**Figure 4:**
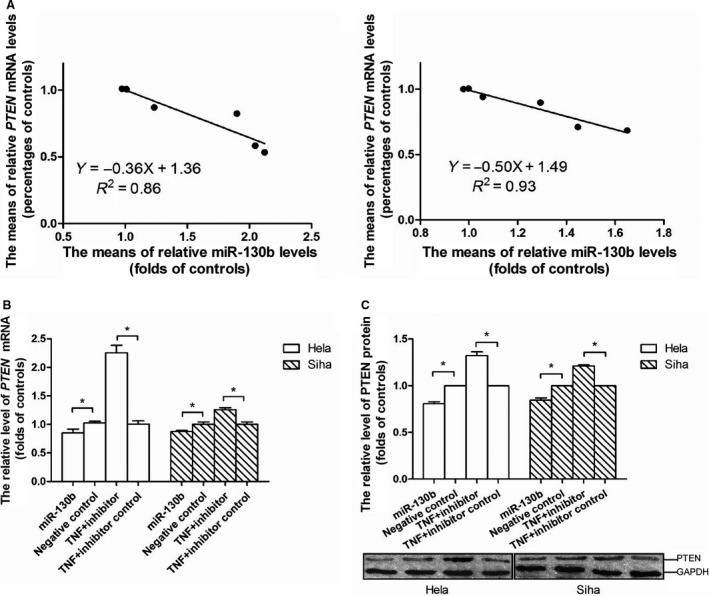
MiR‐130b mediated the TNF‐α‐induced downregulation of *PTEN* gene expression. (A) Regression analyses on the expression levels of miR‐130bs and *PTEN* mRNA showed that there were reverse correlations between miR‐130b and *PTEN* mRNA levels in Hela and Siha cells. The lines plotted in the left and right graphic data represented the relationships between the miR‐130b and *PTEN* mRNA levels in Hela cells and that in Siha cells, respectively. (B) The increments in miR‐130b expression levels resulted in the losses in the *PTEN* mRNA contents in Hela and Siha cells. (C) The increases in miR‐130b expression levels caused the reductions in the PTEN protein contents in Hela and Siha cells. The graphic data obtained in western blot assays located at the bottom of the statistical analyses of the graphic data. Circles represented the relative levels of *PTEN* mRNA or PTEN proteins in TNF‐α‐treated Hela cells and triangles represented the levels in treated Siha cells as compared with the corresponding levels of vehicle‐treated cells. The note ‘miR‐130b’ or ‘negative control’ under the horizontal axis indicated the tumour cell transfected with miR‐130b mimics or their scrambled control oligonucleotides while ‘TNF‐α + inhibitor’ or ‘TNF‐α + inhibitor control’ indicated the cell with the miR‐130b inhibitors or the control oligonucleotides of the inhibitors prior to the TNF‐α treatment. The blank columns represented the relative levels of *PTEN* mRNA or protein levels in Hela cells, and the shadow ones represented the levels in Siha cells. Data were described as means ± SE (*n* = 3). The bars indicated the deviations from means. *P < 0.05; NS, nonsignificant.

The result of the regression analysis showed that the relative expression level of miR‐130b in TNF‐α‐treated cell was inversely correlated with the level of *PTEN* mRNA (Fig. 4A). We carried out the gain‐ and loss‐of‐function assay to test whether there was a causal relationship between the increment in the cellular level of miR‐130b and the reduction in the level of *PTEN* mRNA. The experimental data exhibited that the *PTEN* mRNA level decreased by 17.48% in Hela and by 13.00% in Siha cell which was transfected with miR‐130b mimics in comparison with the corresponding level in the cell transfected with the scrambled control oligonucleotides. After the cell subjected to the treatment of TNF‐α at the concentration of 10 ng·mL^−1^ over a 48‐h time period, the *PTEN* mRNA level increased by 1.24‐fold in Hela and by 0.26‐fold in Siha cell transfected with miR‐130b inhibitors when compared with the counterpart in the cell transfected with the scrambled controls (Fig. 4B). Furthermore, we found there were a 19.14% decrease in the level of PTEN protein in Hela cell and a 15.38% decrease in Siha cell transfected with miR‐130b mimics as compared with the corresponding protein levels in the cells transfected with the scrambled control oligonucleotides. The PTEN protein levels in Hela and Siha cells transfected with miR‐130b inhibitors increased by 0.32‐ and 0.21‐fold as compared with the corresponding levels in cells with the scrambled control oligonucleotides (Fig. 4C).

### MiR‐130b suppressed the expression of PTEN gene by directly base pairing with the 3′UTR of PTEN mRNA

**Figure 5:**
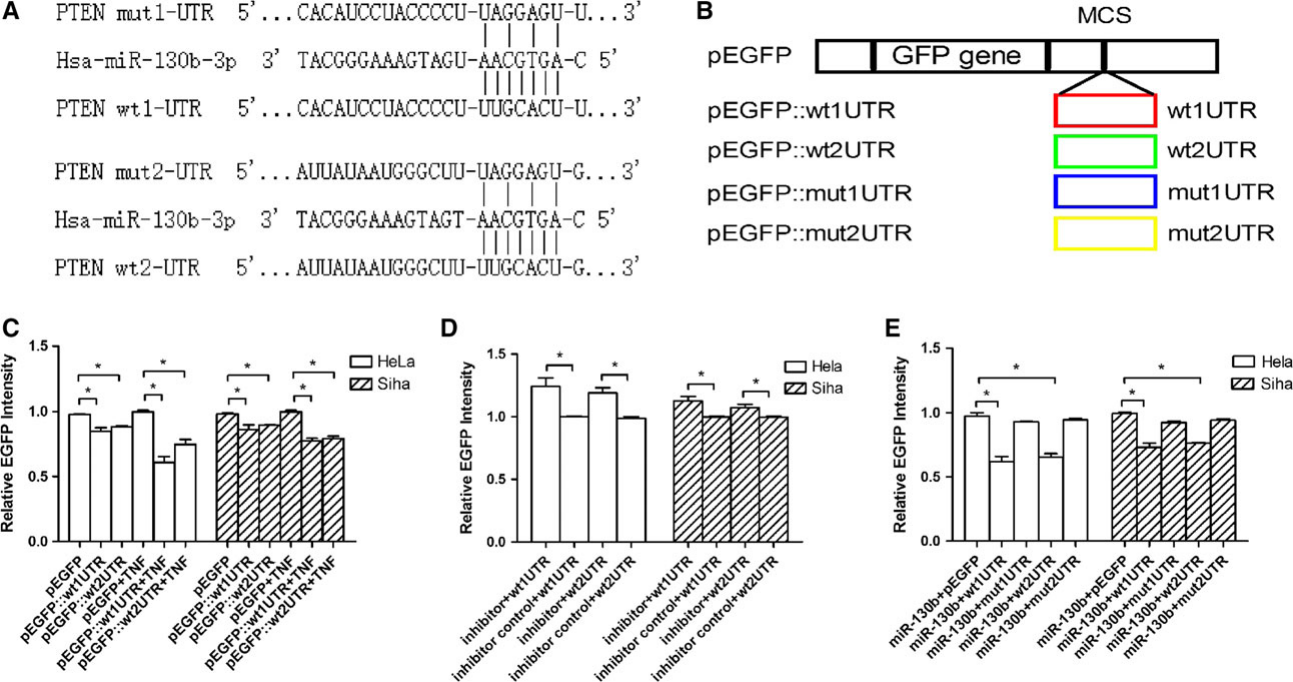
MiR‐130b inhibited the expression of *PTEN* gene by directly binding to the 3′UTR of *PTEN* mRNA. (A) There were two putative miR‐130b targeted sites existing in the 3′UTR of *PTEN* mRNA. The dotted lines indicated the regions of wild‐type or mutated 3′UTRs whose sequences were not shown. The vertical lines indicated the base pairs between the seed sequences of miR‐130b and the regions complementary to the seed sequences within the wild‐type or mutated 3′ UTRs of *PTEN* mRNA. (B) Domain structures of pEGFP and its derived vectors. The block labelled by ‘GFP gene’ represented the coding sequence of GFP in the vector, and the block labelled by ‘wt1/2 UTR’ or ‘mut1/2 UTR’ represented the DNA domain encoding the part of wild‐type or mutated UTR of *PTEN* mRNA. MCS meant multiple cloning sites. (C) The expression of GFP was suppressed in the tumour cell with pEGFP::wt1UTRs or pEGFP::wt2UTRs as compared with the cell with pEGFP control vectors. The note ‘pEGFP’, ‘wt1UTR’ or ‘wt2UTR’ under the horizontal axis represented the tumour cell transfected with pEGFP or its derived vectors, pEGFP::wt1UTRs or pEGFP::wt2UTRs, prior to TNF‐α treatment. (D) MiR‐130b took part in the decrease in the expression of GFP caused by TNF‐α treatment in Hela or Siha cell transfected with pEGFP::wt1UTRs or pEGFP::wt2UTRs. The note ‘inhibitor + wt1/2UTR’ or ‘inhibitor control + wt1/2UTR’ under the horizontal axis represented the tumour cell cotransfected with miR‐130b inhibitors and pEGFP::wt1/2UTRs or miR‐130b inhibitor controls and pEGFP::wt1/2UTRs prior to TNF‐α treatment. Data were described as means ± SE (*n* = 3). The bars indicated the deviations from means. (E) MiR‐130b suppressed the gene expression of GFP by binding to the putative site within the 3′UTR of reporter gene mRNA derived from *PTEN* mRNA in Hela and Siha cell. The note ‘miR‐130b + wt1/2UTR’ or ‘negative control + wt1/2UTR’ under the horizontal axis indicated the tumour cell cotransfected with miR‐130b mimics or their scrambled control oligonucleotides and pEGFP::wt1/2UTRs. The note ‘miR‐130b + mut1/2UTR’ or ‘negative control + mut1/2UTR’ under the horizontal axis indicated the tumour cell cotransfected with miR‐130b mimics or their scrambled controls and pEGFP::mut1/2UTRs. Data were described as means±S. E. (*n* = 3). The bars indicated the deviations from means. *P < 0.05; NS, nonsignificant.

*In silico* analysis revealed that there were two putative targeted sites of miR‐130b existing in the 3′UTR of *PTEN* mRNA (Fig. 5A). To test whether the putative targeted sites were involved in the regulation of *PTEN* expression by miR‐130b, the DNA sequence encoding the UTR encompassing wild‐type or mutated targeted site was cloned into the pEGFP vector downstream of the coding domain of GFP gene (Fig. 5B). The constructed vectors were transfected into Hela or Siha cells, and then, the transfected cells were incubated in the medium containing 10 ng·mL^−1^ TNF‐α over time courses of 48 h. The intensity of the fluorescence emitted by the target cell transfected with pEGFP::wt1‐UTRs and pEGFP::wt2‐UTRs decreased by 38.98% and by 25.05% in Hela cell and decreased by 22.44% and by 20.64% in Siha cell as compared with the intensity of fluorescence produced by the cell with pEGFP control vectors (Fig. 5C). After the target cell exposed to TNF‐α, the transfection of miR‐130b inhibitors increased the intensity of fluorescence from the cell with pEGFP::wt1‐UTRs and pEGFP::wt2‐UTRs by 0.23‐ and 0.21‐fold in Hela cell and by 0.13‐ and 0.08‐fold in Siha cell when compared with the transfection of miR‐130b inhibitor control oligonucleotides (Fig. 5D). Conversely, the transfection of miR‐130b mimics produced the attenuation in the intensity of fluorescence from the cell with pEGFP::wt1‐UTRs and pEGFP::wt2‐UTRs by 36.37% and 32.96% in Hela cell and by 26.69% and 23.68% in Siha cell when compared with the intensity of fluorescence from the cell with pEGFP control vectors, but no significant change was found between the intensity of fluorescence from the cell with pEGFP::mut1‐UTRs or the cell with pEGFP::mut2‐UTRs and the intensity from the cell with pEGFP control vectors in either Hela or Siha cell transfected with miR‐130b mimics (Fig. 5E).

## Discussion

Some specific cell lines are susceptible to toxic actions of TNF‐α, but the vast majority of cell lines develop resistances to TNF‐α cytotoxicity by different mechanisms. In this study, we observed that two cervical cancer cell lines had no significant loss in the cell viabilities after treated with various concentrations of TNF‐α ranging from 0.1 to 50 ng·mL^−1^ when compared with vehicle‐treated cells (Fig. 1A). The data suggested these cervical cancer cells resistant to the toxicities mediated by TNF‐α at these concentrations. While there was no obvious influence of TNF‐α on the viability of tumour cell, the expression level of miR‐130b in target cell mounted in response to the increase in the TNF‐α concentration (Fig. 2A). The recent literatures illustrated that NF‐κB mediated the TNF‐α‐upregulated expressions of miR‐130b in both adipocyte and bladder carcinoma cell lines by directly binding to its response elements within the miR‐130b promoters 21, 22. And it has been generally known that TNF‐α stimulation induced the activation of NF‐κB in Hela cell. Based on these facts, we considered that the TNF‐α‐promoted expression of miR‐130b in cervical cancer cell might be through the activation of NF‐κB signalling pathway. MiR‐130bs have been verified to play oncogenic roles in the developments of different types of cancers. We guessed that the TNF‐α‐stimulated expression of miR‐130b might be involved in the resistance of cervical cancer cell to TNF‐α killing. To demonstrate this assumption, we transfected miR‐130b inhibitors or their scrambled controls into TNF‐α‐ or vehicle‐treated tumour cells. The experimental data showed that blocking endogenous miR‐130b produced a decrease in the viability of vehicle‐treated cervical cancer cell, but more reduction in the viability was observed in TNF‐α‐treated counterpart (Fig. 2B). These results presented that miR‐130b performed an oncogenic function in cervical cancer cell and TNF‐α‐induced expression of miR‐130b contributed to the resistance of target cell to TNF‐α killing. It is well known that TNF‐α exerts its toxicity by inducing apoptosis in a variety of cell types. This process can proceed through both the extrinsic apoptosis and the mitochondria‐mediated pathway 5. To see the impact of TNF‐α on the apoptosis of cervical cancer cell, we incubated the tumour cell with various concentration of TNF‐α but no notable alteration in the apoptosis rate was seen between TNF‐α‐ and vehicle‐treated cell (Fig. 1B). However, the transfection of miR‐130b inhibitors into the tumour cell prior to TNF‐α treatment resulted in the increment in apoptosis rate as compared with the transfection of the scrambled controls of inhibitors (Fig. 2C). These facts indicated that the TNF‐α‐enhanced expression of miR‐130b protected the tumour cell from the apoptosis induced by TNF‐α.

The PTEN protein is characterized as a dual‐specificity phosphatase and known as a famous tumour suppressor. It modulates cell growth and survival by negatively regulating the phosphoinositide 3‐kinase (PI3K)/Akt signalling pathway 24, 25. In the recent decades, the researchers found that the miRNA‐regulated PTEN/PI3K/Akt signalling pathway was associated with the drug resistance of tumour cell. MiRNA‐21 induced gemcitabine resistance by suppressing the expression of *PTEN* gene in breast cancer 26. MiR‐221 attenuated the sensitivity of cervical cancer cell to gefitinib through downregulating the *PTEN* gene expression 27. MiR‐106 triggered cell radioresistance via inhibiting the expression of *PTEN* gene in colorectal cancer 28. In our study, we discovered that TNF‐α stimulation brought about the attenuation in the level of *PTEN* mRNA as well as protein in cervical cancer cell. Although TNF‐α treatment did not cause obvious changes in the viability and apoptosis rate of tumour cell, the overexpression of exogenous *PTEN* gene in tumour cell produced a reduction in the viability and an elevation in the apoptosis rate of target cell after exposed to TNF‐α treatment (Fig. 3A–D). These experimental data suggested that cervical cancer cells should develop their resistances to TNF‐α cytotoxicity by suppressing the expression of *PTEN* gene.

Regression analysis showed that there was an inverse relationship between miR‐130b and *PTEN* mRNA level in cancer cell (Fig. 4A). The transfection of miR‐130b inhibitors antagonized the TNF‐α‐induced downregulation of *PTEN* mRNA and protein levels, whereas the transfection of miR‐130b mimics caused decreases in the cellular levels of both *PTEN* mRNA and protein (Fig. 4B,C). These evidences presented that there was a causal relationship between the accumulation in miR‐130b and the reduction in *PTEN* mRNA or protein level in cervical cancer cell and supported that miR‐130b mediated the TNF‐α‐stimulated inhibition of *PTEN* gene expression.

The intensity of fluorescence from the cell with either pEGFP::wt1‐3′UTRs or pEGFP::wt2‐3′UTRs was lower than the intensity from the cell with pEGFP control vectors (Fig. 5C). This indicated that the 3′UTR region was associated with the downregulation of reporter gene in target cell, and the more loss in the intensity of fluorescence from TNF‐α‐treated cell than that from vehicle‐treated cell showed that the 3′UTR region might be involved in the TNF‐α‐induced suppression of reporter gene expression. As it was proved that TNF‐α stimulation increased the level of miR‐130b in cervical cancer cell, we assumed that miR‐130b must participate in the regulation of reporter gene. And this assumption was supported by the fact that blocking miR‐130b function resulted in an increase in the intensity of fluorescence from TNF‐α‐treated cell with either pEGFP::wt1‐3′UTRs or pEGFP::wt2‐3′UTRs (Fig. 5D). MiRNA regulate gene expression through complementary binding to 3′UTR region of targeted mRNA 29. Two targeted sites of miR‐130b were predicated to locate within the 3′UTR of *PTEN* mRNA, and the transfection of exogenous miR‐130b caused an apparent reduction in the expression of reporter gene in the cell containing the pEGFP::wt1‐3′UTRs or pEGFP::wt2‐3′UTRs but not in the cell containing pEGFP::mut1‐3′UTRs or pEGFP::mut2‐3′UTRs (Fig. 5A,E). These evidences suggested that miR‐130b should inhibit the expression of its target gene based on the base pairing between the seed sequence of miR‐130b and its complementary region within the 3′UTR of targeted mRNA.

## Author contributions

HM offered the idea for the research. LY was responsible to design the experiments for the research and participated in the experiments. YW and SS were involved in the experiments and responsible for the analysis of experimental data obtained in the research. LX, TL and YW were involved in the experiments.
